# Peptide Extract from Red Kidney Beans, *Phaseolus vulgaris* (Fabaceae), Shows Promising Antimicrobial, Antibiofilm, and Quorum Sensing Inhibitory Effects

**DOI:** 10.1155/2024/4667379

**Published:** 2024-04-04

**Authors:** Jennifer Tetteh, De-Youngster Wereko Brobbey, Kofi Junior Osei, Azumah Ayamah, Michael Konney Laryea, Godfred Darko, Lawrence Sheringham Borquaye

**Affiliations:** ^1^Department of Chemistry, Kwame Nkrumah University of Science and Technology, Kumasi, Ghana; ^2^Department of Crop and Soil Science, Kwame Nkrumah University of Science and Technology, Kumasi, Ghana; ^3^Central Laboratory, Kwame Nkrumah University of Science and Technology, Kumasi, Ghana

## Abstract

The rapid spread of multidrug-resistant bacteria has led to an increased risk of infectious diseases. *Pseudomonas aeruginosa*, in particular, poses a significant obstacle due to its propensity to rapidly acquire resistance to conventional antibiotics. This has resulted in an urgent need for the development of new classes of antibiotics that do not induce resistance. Antimicrobial peptides (AMPs) have been studied as potential small-molecule antibiotics due to their unique mode of action. In this study, peptides were extracted from the seeds of *Phaseolus vulgaris* (Fabaceae), and the antimicrobial activities of the extract were evaluated using microbroth dilution against five different microorganisms. The extract showed antimicrobial activity against all tested organisms with minimum inhibitory concentrations (MIC) of 2.5 mg/mL, except for *Candida albicans* and *Pseudomonas aeruginosa*, which had MICs of 1.25 mg/mL. The extract was also bacteriostatic for all tested organisms. The crude peptide extract from *Phaseolus vulgaris* was further studied for its antibiofilm activity against *Pseudomonas aeruginosa*, a common nosocomial pathogen associated with biofilm formation. The extract showed good antibiofilm activity at 1/2 MIC. The extract also inhibited the expression of pyocyanin and pyoverdine (virulence factors of *P. aeruginosa* whose expression is mediated by quorum sensing) by 82% and 66%, respectively. These results suggest that the peptide mix from *Phaseolus vulgaris* may inhibit biofilm formation and virulence factor expression by interfering with cell-to-cell communication in *Pseudomonas aeruginosa*. The ability of the extract to inhibit the growth and biofilm formation of all tested organisms indicates its potential as an antimicrobial agent that could be further studied for drug discovery.

## 1. Introduction

Despite advancements, infectious diseases remain a global health challenge. Pathogenic organisms like bacteria, fungi, parasites, and viruses cause infectious diseases. Microbial pathogens are responsible for over 400 million years of life lost annually, surpassing cancer and cardiovascular diseases. Antibiotics have demonstrated effectiveness in the treatment of infections by targeting pathogens while causing minimal harm to host cells. They have saved countless lives and are vital in treating various infections and life-threatening conditions. However, bacterial resistance has risen due to antibiotic overuse and limited development of new antibiotics. Multi-drug-resistant (MDR) and extremely drug-resistant (XDR) bacteria have emerged, posing a major global threat [1]. Projections indicate that antibiotic resistance could lead to 10 million annual deaths globally and economic costs of $100 trillion by 2050. It also hinders universal health coverage and the attainment of some of the sustainable development goals. Addressing antimicrobial resistance and developing new treatments are critical for global health progress [2].

Pathogenic microorganisms employ diverse mechanisms to develop resistance against antibacterial agents, including efflux pumps, target modification, and the formation of biofilms [3]. *Pseudomonas aeruginosa* is a model bacterium known for its proficiency in biofilm formation and its role as an opportunistic pathogen in cystic fibrosis-related respiratory infections, as well as chronic infections in immunocompromised individuals [4]. Additionally, *P. aeruginosa* is associated with tissue degradation and spoilage of meat and protein-rich foods [5]. The formation of biofilms in *P. aeruginosa* is intricately regulated by quorum sensing (QS). QS serves as a cell-to-cell communication system that governs the expression of genes related to biofilm formation. Studies have underscored that *P. aeruginosa* strains with defective QS mechanisms are unable to form biofilms. Moreover, QS has been shown to exert a significant impact on the expression of various virulence factors in *P. aeruginosa*, including pyoverdine, pyocyanin, and proteases. Two interconnected QS systems in *P aeruginosa*, Las and Rhl, are of utmost importance for virulence and biofilm production, as well as tolerance to antibiotics, detergents, and biocides [6]. The Las system, under the guidance of LasR, governs other QS systems and influences biofilm formation and the activation of virulence genes. LasI, within this system, produces N-(3-oxododecanoyl) homoserine lactone (3-O-C12-HSL), which triggers LasR to activate virulence genes, such as lasB, lasA, apr, and toxA [7]. Two additional QS systems, namely, Pseudomonas quinolone signal (PQS) and integrated quorum system (IQS), exist, with the latter's specifics yet to be fully elucidated [8]. The Las system emerges as a central player in QS, with LasR orchestrating various systems and genes critical for the bacterium's behavior [9].

One promising avenue for finding novel antibacterial remedies lies in the exploration of natural products that are commonly consumed. Antimicrobial peptides (AMPs) have emerged as a focal point of study, and applications in both agriculture and medicine have been touted [10]. AMPs are a widespread class of molecules found in diverse organisms, ranging from microbes to animals, and are key components of natural defense systems against pathogens and pests. While AMPs come in different molecular forms, most of them are linear peptides derived from insects, animals, and plants. Plants, in particular, possess an extensive distribution of AMPs, which can be extracted and isolated from various plant organs, such as stems, roots, seeds, flowers, and leaves. These AMPs exhibit diverse physiological defense mechanisms against viruses, bacteria, fungi, and parasites, making them potential candidates for therapeutic and preservative applications [11].

Generally, AMPs derived from plants share several common features with those obtained from microorganisms, insects, and animals. Some of these features include the molecular form they exhibit, the overall positive charge of the peptide, and their amphipathic nature. All of these characteristics play a critical role in their function as defensive agents, acting as membrane-active antimicrobials. Two plant AMP families that exhibit these features are thionins and plant defensins. In addition to these characteristics, both thionins and plant defensins are notably rich in cysteine residues. It is worth noting that other plant AMP families typically exert their antimicrobial effects on pathogenic microbes in a manner distinct from AMPs derived from animals [12]. As an example, hevein-like peptides are known to function by binding to chitins, whereas knottin-type peptides play a critical role in inhibiting key enzymes, such as proteases. Lipid transfer peptides interact strongly with anionic lipopolysaccharides found in the outer membranes of many Gram-negative bacteria (such as *P. aeruginosa*), leading to destabilization and permeabilization of the membrane [12]. The expression of AMP in plants is induced and usually tissue-specific, just as it is observed in animals. Once more, plant-derived AMPs exhibit adaptability through the presence of highly variable sequences housed within a specific scaffold unique to each AMP family. This phenomenon bears some resemblance, albeit on a considerably smaller scale, to the molecular diversity observed in the immune system's immunoglobulin-based immunity among vertebrates [12].

Red kidney beans, known as *Phaseolus vulgaris* (Fabaceae), have garnered increasing interest in recent research. These beans are recognized for their positive health benefits. Rich in amino acids, red kidney beans offer a cost-effective source of dietary protein, typically comprising 20% to 30% protein content based on dry weight. Previous investigations on peptides isolated from related species have demonstrated various biological activities [10, 13]. Antimicrobial protein hydrolysate was isolated from *P. vulgaris,* which is active against some Gram-positive and Gram-negative bacteria [14], setting a foundation for exploring the antimicrobial potential of crude peptides from red kidney beans. In this study, the antimicrobial, biofilm inhibition, and quorum sensing effects' crude peptides extracted from *Phaseolus vulgaris* were investigated, contributing to their application on health in addition to their nutritional values.

## 2. Methods

### 2.1. Sample Collection

**Figure 1:**
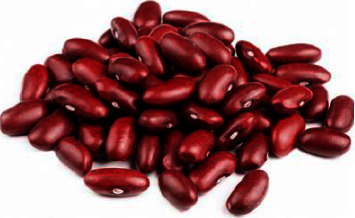
Red kidney beans (*P. vulgaris*).

Red kidney beans (Figure 1) were collected from the local market of Ayeduase, Kumasi, in the Ashanti Region of Ghana and helped verified by a plant botanist at the Department of Herbal Medicine, Kwame Nkrumah University of Science and Technology. Beans with uniform size and shape were selected for this work. The beans were air-dried and stored in a cool-dried container at room temperature (27°C) until further analysis.

### 2.2. Peptide Extraction

Crude peptide was extracted from red kidney beans using method described by Roy and coworkers [13] with slight modification. The beans were first grounded into flour, after which the resulting flour was mixed with distilled water in a ratio of 1 : 10 (w/v). The pH of the mixture was then adjusted to 9.0 using 1N NaOH and stirred at room temperature (27°C) for 2 hours. The mixture was then centrifuged at 6000 rpm for 20 minutes, and the precipitates were discarded. The supernatant was acidified to a pH of 4.5 using 1N HCl and centrifuged to be obtained the precipitates. The resulting precipitates were washed twice with distilled water at a ratio of 1 : 5 (w/w). After washing, the obtained precipitates were freeze-dried to obtain the crude peptide extracts.

### 2.3. Characterization

#### 2.3.1. Infrared Spectroscopy

The infrared spectrum of the peptide was determined on a Fourier Transform Infrared (FTIR) equipment (Platinum-ATR, Bruker, Germany). Dried extracts obtained from lyophilization were used. The sample was introduced onto the crystal plate and was scanned from 200 to 4000 nm^−1^ wavenumber, for twenty-four times to obtain the spectrum of the reference standards.

#### 2.3.2. UV-Vis Spectroscopy

The absorbance of the peptide was measured using a Specord®200 Plus UV-Vis equipment (Analytik Jena, Germany). The scanning was performed in the range of 185 nm to 320 nm. The dried extract obtained from lyophilization was redissolved in 0.1% trifluoroacetic acid (TFA) in 25% acetonitrile, which was used for this analysis.

#### 2.3.3. Biuret Test

The presence of peptide bonds was confirmed by performing a biuret test. The peptide extract was reconstituted in 0.1% TFA in 25% acetonitrile. 5% NaOH solution was added followed by few drops of 1% copper sulfate solution. A change in color from pale blue to purple indicated the presence of peptide bonds [15].

### 2.4. Antimicrobial Assays

#### 2.4.1. Microbial Cultures

The antimicrobial activities of the crude peptide extracts were tested against laboratory strains of Gram-positive bacteria (*Enterococcus faecalis* ATCC 29212 and *Staphylococcus aureus* ATCC 25923), Gram-negative bacteria (*Pseudomonas aeruginosa* ATCC 4853 and *Escherichia coli* ATCC 25922), and a fungus (*Candida albicans* ATCC 10231). The microbial strains used in this study were sourced from the Department of Pharmaceutical Microbiology, KNUST.

#### 2.4.2. Inoculum Preparation

The microbial isolates were streaked onto nutrient agar plates (Oxoid, UK) and incubated at 37°C for 18–20 hours to promote growth. Subsequently, subcultures were obtained and used to prepare colony suspensions of the microorganisms, which were then adjusted to a 0.5 McFarland standard. These colony suspensions were further diluted into sterile double-strength CM001 nutrient broth (Oxoid, UK), resulting in a concentration of approximately 2 × 10^5^ CFU/mL.

#### 2.4.3. Minimum Inhibitory Concentration (MIC)

The minimum inhibitory concentration (MIC) of the peptide extract was determined by the broth microdilution method [16]. Eight serial twofold dilutions of peptide extract were prepared, resulting in a concentration range of 2.5 to 1.953 × 10^−3^ mg/mL. Gentamicin and fluconazole were used as the positive controls with concentrations ranging from 50 to 1.96 × 10^−1^ *μ*g/mL for bacterial and fungal strains, respectively. Each well of the microtiter plate was filled with 40 *μ*L of double-strength broth containing an inoculum size of 2 × 10^5^ CFU/mL. The final volume in each well was 200 *μ*L. The plates were then covered and incubated at 37°C for 24 hours.

#### 2.4.4. Minimum Bactericidal Concentration (MBC)

MBC/MFC values were determined by pipetting 40 *μ*L of microbial suspension from subculture demonstrating no visible growth from the MIC experiment and inoculating nutrient agar plates. The MBC or MFC was determined with the wells with concentrations greater than the MIC. Plates were incubated at 37°C for a total period of 24 hours. Each experiment was done in triplicate. Minimum bactericidal concentration (MBC) and minimum fungicidal concentration (MFC) were recorded as the lowest extract concentration killing 99.9% of the bacterial or fungal inocula after 24-h incubation at 37°C [3].

*Evaluation of Microbicidal and Microbistatic Capacity of Peptide Extract*. The antimicrobial activity of the compounds was assessed using the MBC (or MFC)/MIC ratio. An MBC (or MFC)/MIC ratio ≤2 indicated a microbicidal effect, while a microbistatic effect was recorded when the MBC (or MFC)/MIC ratio was ≥4 [17].

### 2.5. Biofilm Inhibition Assay

The biofilm inhibition capacity of the peptide extract was determined using the crystal violet assay [18]. Sterile microtiter plates were filled with both MIC and sub-MIC concentrations of the peptide extract and a standard drug (gentamicin). Inoculum suspensions adjusted to 0.5 McFarland standard were added to each well, resulting in a final volume of 200 *μ*L. The plates were incubated at 37°C for 24 hours. After incubation, the contents of the wells were discarded and thoroughly washed with deionized water. This was to remove any unbound or loosely attached bacterial cells. The wells were stained with 0.1% crystal violet, and the contents were eluted with 30% acetic acid onto a new sterile plate. The absorbance of the eluates was measured at 595 nm, allowing for the evaluation of biofilm inhibition using the following equation:

(1) $$\begin{array}{c} \%\text{ Inhibition}=\frac{\text{control}-\text{treated}}{\text{control}}\times 100\%. \end{array}$$

### 2.6. Inhibition of Quorum Sensing in *P. aeruginosa*

#### 2.6.1. Pyoverdine Quantification

An overnight culture of *P. aeruginosa* (50 *μ*L) was inoculated into 2 mL of nutrient broth in the absence (growth control) and presence of sub-MIC (1/2 MIC, 1/4 MIC, 1/8 MIC, 1/16 MIC, and 1/32 MIC) doses of gentamicin and peptide extract (2 mL) and incubated for 24 hours at 37°C. After incubation, the culture media was then centrifuged at 5000 rpm for 45 minutes. A 96-well microtiter plate was prepared by filling each well with 100 *μ*L of the cell-free supernatant for pyoverdine measurement. The relative concentration of pyoverdine in all treated supernatants with respect to control (growth control) was measured by fluorescence (BioTek® Synergy H1 Multimode Microplate Reader, Germany) at an excitation wavelength of 405 nm and an emission wavelength of 465 nm [19]. The inhibition percentages were determined by comparing the treated cultures to the untreated culture (control) using the following equation:

(2) $$\begin{array}{c} \%\text{ Inhibition}=\frac{\text{control}-\text{treated}}{\text{control}}\times 100\%. \end{array}$$

#### 2.6.2. Pyocyanin Quantification

*P. aeruginosa* was incubated as described in the pyoverdine inhibition assay. Cell-free supernatants were obtained by centrifuging the culture at 4000 rpm for 45 minutes. To 8 mL of the supernatant, 4 mL of chloroform was added, and the mixture was vortexed 10 times for 2 seconds each to separate the green-blue chloroform layer, which settled at the bottom of the tube. The samples were then centrifuged for 2 minutes at 4000 rpm, and the supernatant above the green-blue chloroform layer was carefully removed. Next, 3 mL of 0.2 M HCl was added to each tube, and the mixture was vortexed again 10 times for 2 seconds. After centrifugation for 2 minutes at 4000 rpm, the supernatant (pink layer) was transferred into a cuvette, and the absorbance was measured at 520 nm. The concentration of pyocyanin (*μ*g/mL) was determined by multiplying the absorbance value at 520 nm by 17.072 and the molar extinction coefficient of pyocyanin at 520 nm [3]. Percentage inhibition was calculated relative to the untreated culture (control) using the following expression:

(3) $$\begin{array}{c} \%\text{ Inhibition}=\frac{\text{control}-\text{treated}}{\text{control}}\times 100\%. \end{array}$$

### 2.7. Bacterial Growth Curve

To evaluate the effect of peptides at MIC and sub-MIC levels on the growth of *P. aeruginosa*, optical density (OD) measurements were used. Serial dilutions of the peptides and bacteria at an initial concentration of 2 × 10^5^ CFU/mL were prepared in a 96-well microplate. The microplates were then incubated at 37°C with intermittent shaking every 3 seconds. OD measurements at a wavelength of 600 nm were taken hourly for a period of 24 hours to monitor the growth of *P. aeruginosa* [20].

### 2.8. Data Analysis

All the tests were done in triplicate. The experimental were analyzed using GraphPad Prism Version 6.0 for Windows (GraphPad Software, San Diego, CA, USA) and Microsoft Excel 2007.

## 3. Results

### 3.1. Peptide Characterization

**Figure 2:**
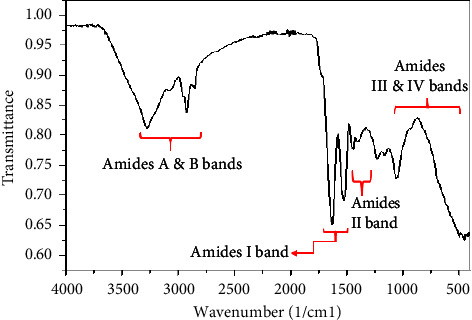
Fourier transform infrared (FTIR) spectrum of peptide extract of *Phaseolus vulgaris*. Amides A and B bands span 3100–3500 cm^−1^, Amide I band is from 1600 to 1700 cm^−1^, Amide II band is from 1480 to 1600 cm^−1^, and the region from 500 to 1300 cm^−1^ represents Amides III-VI bands.

The peptide obtained after extraction and lyophilization was brown in color, with a yield of 35.7%. The dried extracts obtained from lyophilization were used in all the tests. The FTIR spectrum exhibited distinct peaks characteristic of a typical peptide, displaying noticeable bending and stretching vibrations of N-H, C=O, and C-H (Figure 2).

**Figure 3:**
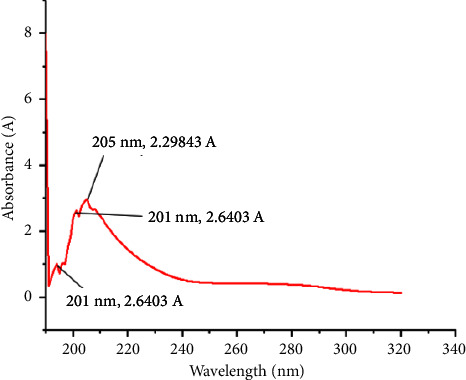
Ultraviolet-visible wavelength of absorption spectrum of peptide extract using ultraviolet-visible spectroscopy. Maximum absorbance (1.2193 Å) occurred at 203 nm and a lower shoulder at 194 nm (0.052 Å).

In the UV characterization of the peptides, the peptide solution demonstrated a maximum absorbance of 1.219 A at 203 nm, accompanied by a minor shoulder at 194 nm (0.075 A), and a weaker band at 280 nm (Figure 3). The peptide extract showed positive results for the biuret test. The formation of purple color after the addition of copper sulfate indicated the presence of peptide bonds in the extract.

### 3.2. Antimicrobial Activity

Peptide extracts derived from *P. vulgaris* displayed good antimicrobial activity, as evidenced by their low minimum inhibitory concentrations (MICs) against Gram-positive and Gram-negative microorganisms. *C. albicans*, a fungus, and *P. aeruginosa*, a Gram-negative bacterium, displayed particularly notable susceptibility, with MICs recorded at 1.25 mg/mL. Additionally, for the Gram-negative bacterium *E. coli* and the Gram-positive bacteria *S. aureus* and *E. faecalis*, the MICs were observed to be 2.5 mg/mL (Table 1). The minimum bactericidal concentration (MBC) and the minimum fungicidal concentration (MFC) of the peptide extract against all tested microbes exceeded 2.5 mg/mL, indicating that higher concentrations were required to achieve microbicidal activity (Table 1). Furthermore, the MBC/MIC and MFC/MIC ratio for all organisms was greater than 1, suggesting a microbistatic effect of the peptide extract. This implies that the peptide extract inhibited the growth of the organisms without completely eradicating them.

### 3.3. Effect of Crude Peptide on Biofilm Formation

**Figure 4:**
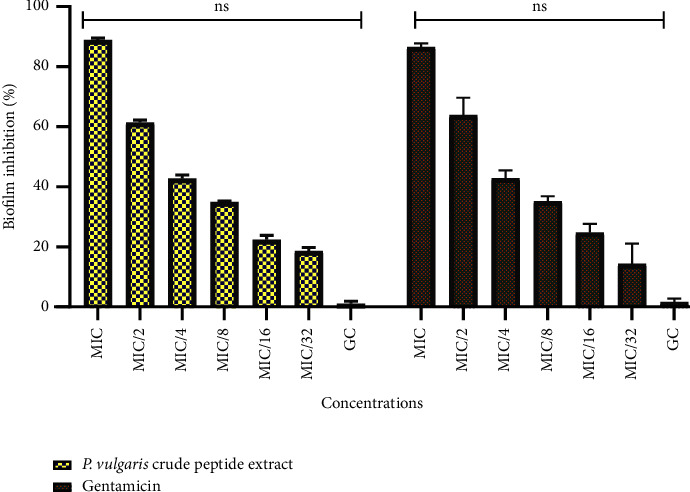
Antibiofilm effect of crude peptide and gentamicin on *P. aeruginosa*. Mean values of three independent experiments and their standard deviations are shown. When compared with the untreated wells (GC), no significant difference was observed.

The antibiofilm activity of the peptide extract was tested against the wild type, widely used biofilm-forming clinical isolate *P. aeruginosa* PAO1. Gentamicin was used as a positive control. Optical density (600 nm) measurements in treated cultures showed that biofilm formation was reduced in a largely dose-dependent manner (Figure 4). At 1/2 MIC and 1/32 MIC, gentamicin inhibited biofilm formation by 64% and 13%, respectively (Figure 4), whereas at the MIC of the peptide extract, biofilm formation was inhibited to about 87% (Figure 4). This reduced to 42% at 1/4 MIC and dropped to 18% at 1/32 MIC. A high inhibition rate was shown at MIC/2 (62%).

### 3.4. Effect of Crude Peptide on Secretion and Inhibition of Virulence Factors

The effect of the peptide extracts in inhibiting quorum sensing (QS) in *P. aeruginosa* was evaluated in the PAO1 strain. As QS regulates the expression of genes involved in the production and secretion of virulence factors in *P. aeruginosa*, the ability of the peptide extract in interfering with the production of pyoverdine and pyocyanin by *P. aeruginosa* was investigated. Again, gentamicin was used as a positive control.

**Figure 5:**
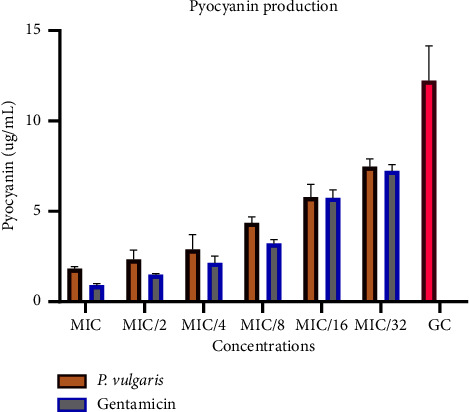
Pyocyanin inhibition. Relative fluorescence of pyocyanin secreted by *Pseudomonas aeruginosa* with and without sub-MIC doses of the peptide extract and standard drug, gentamicin. Each bar represents the mean ± SD of fluorescence intensities of three independent.

**Figure 6:**
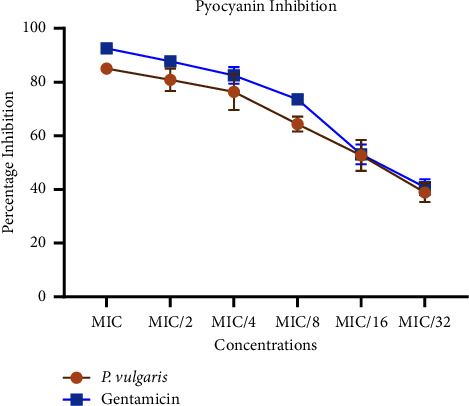
Percentage inhibition of pyocyanin secretion in *Pseudomonas aeruginosa* in the presence of sub-MIC doses of the crude peptides and gentamicin. Percentage inhibitions were computed with respect to the fluorescence of the control group. Each bar represents mean ± SD of triplicate experiments.

The crude peptide extract demonstrated significant anti-QS activity, effectively inhibiting the production of pyoverdine and pyocyanin in a dose-dependent manner at both MIC and sub-MIC concentrations. The levels of pyocyanin produced varied inversely with the concentration of the peptide extract or standard gentamicin used (Figure 5). In the absence of any treatment, the average pyocyanin production in the growth control was 12 *μ*g/mL. At the lowest concentration of the extract or drug, pyocyanin production decreased to approximately 7 *μ*g/mL, further reducing to around 1 *μ*g/mL and 2 *μ*g/mL, respectively, for gentamicin and the peptide extract at their respective MIC concentrations. Percentage inhibition analysis revealed that the peptide extracts inhibited pyocyanin production by 81%, 76%, 64%, 53%, and 41% at sub-MIC concentrations of 1/2, 1/4, 1/8, 1/16, and 1/32, respectively. Notably, at 1/16 and 1/32 MICs, both the extract and standard gentamicin exhibited similar levels of pyocyanin inhibition. The corresponding percentage inhibitions are graphically presented in Figure 6.

**Figure 7:**
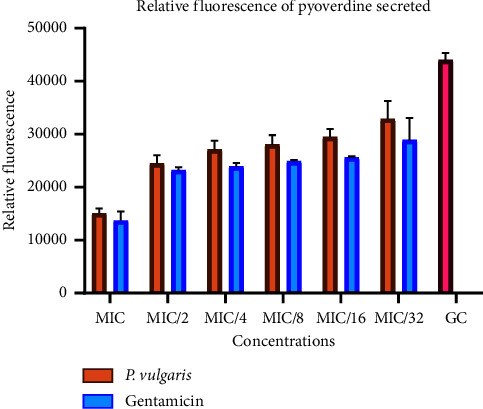
Percentage inhibition of pyoverdine secretion in *Pseudomonas aeruginosa* in the presence of sub-MIC doses of the crude peptides and gentamicin. Percentage inhibitions were computed with respect to the fluorescence of the control group. Each bar represents mean ± SD of triplicate experiments.

**Figure 8:**
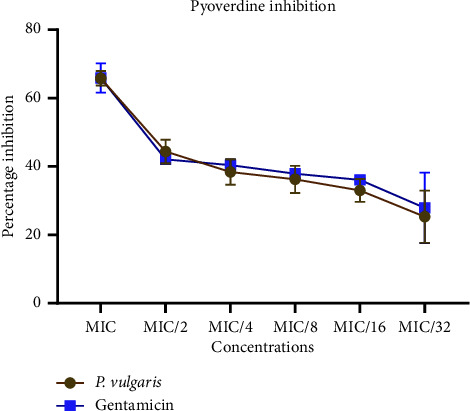
Percentage inhibition of pyoverdine secretion in *Pseudomonas aeruginosa* in the presence of sub-MIC doses of the crude peptide extracts and gentamicin. Percentage inhibitions were computed with respect to the control group. Each bar represents mean ± SD of triplicate experiments.

Regarding pyoverdine, fluorescence emissions demonstrated a dose-dependent relationship, with significantly lower fluorescence in the treated groups compared to the growth control (Figure 7). Both gentamicin and the peptide extract exhibited similar inhibitory effects on pyoverdine production at various concentrations. At the MIC, pyoverdine production was maximally inhibited by 65%. The inhibition of pyoverdine between MIC/2 and MIC/32 remained relatively consistent, ranging between 44% and 25% for both the gentamicin and peptide extract treatments (Figure 8).

Assessing the growth rate of *Pseudomonas aeruginosa* in the presence of MIC and sub-MIC concentrations of the peptide extract, a marginal decrease was observed compared to the growth control. While the MIC treatment resulted in no bacterial growth, as evidenced by consistent OD measurements over 24 hours, the sub-MIC treatments followed a growth pattern similar to that of the control, albeit at a slower rate during the lag phase. The impact of the peptide extract on the growth rate of *P. aeruginosa* was concentration-dependent.

## 4. Discussion

*Phaseolus vulgaris* (Figure 1), an essential legume with its roots in Central and South America, holds great significance in human nutrition. It has achieved global dietary prominence due to its nutrient-rich composition and versatility in cooking. Beyond being a protein source, it provides vital vitamins, minerals, and dietary fiber, contributing to sustainable agriculture and addressing global nutrition challenges [10, 14]. The extraction of antimicrobial peptides (AMPs) can be performed from various parts of the plant. In this study, peptides were extracted from the seeds, as they represent the proteinaceous component of the plant.

To enhance peptide stability and minimize degradation during the extraction process, the pH of the seed slurry was adjusted to 9.0. This higher pH value is known to promote peptide stability and reduce the risk of degradation or hydrolysis [21]. Precipitates were obtained by further adjusting the pH to 5.0 as the peptides may exhibit reduced solubility due to alterations in intermolecular forces, such as hydrophobic interactions or hydrogen bonding, which impact their solubilization in the surrounding solution [22]. Thus, adjusting the pH to 5.0 may induce changes in these intermolecular forces, resulting in decreased peptide solubility and subsequent precipitation.

Following peptide precipitation, the biuret test was used to confirm the presence of peptides in the precipitate. The biuret test is a chemical assay used to detect the presence of peptide bonds in a substance. It relies on the biuret reaction, where a substance containing at least two peptide links turns violet when treated with alkaline copper sulfate. In an alkaline solution, a blue-colored Cu^2+^ forms a complex with peptide bonds due to unshared electron pairs on nitrogen and oxygen. This complexation occurs between Cu^2+^ ions and the carbonyl oxygen (>C=O) and amide nitrogen (=NH) of the peptide bond, changing the solution from blue to purple. The depth of purple indicates the quantity of peptide–copper complexes, and the reaction applies to compounds with at least two H_2_N-C, H_2_N-CH_2_-, H_2_N-CS-, or similar groups directly linked or via a carbon or nitrogen atom. Each copper ion likely forms coordinate bonds with six nearby peptide linkages. The color intensity correlates with the number of peptide bonds in the reacting protein molecule and the quantity of protein molecules in the system [15]. Thus, the precipitate obtained from the seeds of *P. vulgaris* was found to be peptide-rich. Mim employed the biuret test in determining the presence of amino acids (peptide bonds) in wool and limed hair [23]. Valdoz also determined the presence of peptides in coconut and macapuno fruits using the biuret test [24]. The presence of peptides in both samples in each analysis was confirmed by the violet color observed after the analysis.

The extract's FTIR spectrum (Figure 2) exhibited consistent vibrational spectra with reported peptide spectra. Peptides display various vibrational frequencies, with the amide I and amide II bands being prominent in the peptide IR spectrum. The amide I region, spanning from 1,700 to 1,600 cm^−1^, is particularly responsive in detecting secondary structure compositions. It arises from the stretching vibration of the C=O bond in the amide group, coupled with the in-phase bending of the N-H bond and stretching of the C-N bond. Each frequency within this range corresponds to a particular protein structure. The amide II band, which is more complex than amide I, primarily originates from in-plane N-H bending and C-N stretching vibrations. While it is conformationally sensitive, it has been less commonly utilized for protein structure analysis. Other amide bands have limited utility in protein structure analysis due to their complexity and their dependence on factors, such as the force field, side chain nature, and hydrogen bonding [25]. The FTIR spectrum of the peptide obtained was consistent with other spectrums reported in literature [3, 20, 26, 27]. The similarities in vibrational stretches observed in both the reported works, and the spectrum obtained from this study confirms that the extract obtained confirms that the extract is peptidic.

Peptides possess inherent chromophores that exhibit various responses to UV light [28]. The electronic absorption spectra of peptides are commonly investigated within the ultraviolet range of the electromagnetic spectrum, spanning from 185 nm to 320 nm. An attenuated absorption at 194 nm was detected (Figure 2), indicating the presence of peptide bonds within the extract. The absorption at 194 nm is a characteristic *π*⟶*π*^*∗*^ of the peptide backbone, aiding in peptide bond identification. Additionally, a prominent absorption peak at 203 nm, corresponding to peptide bonds, was also observed. The peak at 203 nm corresponds to a *n*⟶*π*^*∗*^ transition of the peptide bond, providing a distinct spectral marker for peptide bond identification [11, 29, 30]. Similar absorptions at 194 nm and 240 nm of peptide extract were reported by Gasu and coworkers [26], indicating the presence of peptides in the extract. Again, Wang and his research team observed UV absorptions at 191 nm in a solution of oyster protein hydrolysate and deduced that these absorptions originated from the peptide linkages present within the hydrolysate [31].

Antimicrobial peptides (AMPs) are often effective against a wide range of microorganisms and are considered a potential solution to combat antimicrobial resistance. Since AMPs primarily target cell membranes, any resistance from microorganisms would likely require significant and costly modifications to their entire lipid membrane structure. Consequently, AMPs offer a promising therapeutic choice as such modifications would be challenging for microorganisms to achieve [32]. The susceptibility of the microorganisms was evaluated using the broth microdilution method, which is considered more sensitive compared to other assays for screening antimicrobial natural products [16, 33]. A total of five microbial strains, consisting of two Gram-positive bacteria, two Gram-negative bacteria, and a fungus, were examined. The MICs at which the tested organisms are inhibited and the distributions of MICs for these organisms are summarized in Table 1. Peptide extract obtained from *P. vulgaris* displayed antimicrobial activity against both Gram-positive and Gram-negative bacteria, as well as a fungus. The extract showed low MIC values for all tested organisms, particularly against *C. albicans* and *P. aeruginosa*, indicating its broad-spectrum antimicrobial activity [33]. In general, the MIC values recorded are much lower than those recorded for peptide extracts from *Olivancillaria hiatula* [20], *Patella rustica,* and *Galatea paradoxa* [34], as well as peptide extracts from an Australian plant mixture [35]. These results are consistent with the research conducted by Sarnthima and Khanmmuang [28], who also reported significant MIC values when testing *S. stramoniifolium* seed extracts against *P. aeruginosa*. Furthermore, the MIC values obtained in this study fall within the same range as those recorded for pexiganan, an antimicrobial peptide that has reached advanced stages of clinical trials for treating diabetic foot ulcers [36, 37].

The antimicrobial activity of the peptide extracts from *P. vulgaris* can be attributed to their capacity to disrupt bacterial membranes by interacting with lipid molecules on the cell surface of the tested microorganisms, as is observed for many AMPs [12, 26]. This interaction is facilitated by the linear *α*-helical structure present in the peptide extract. Antimicrobial activity has been shown in several cases to correlate closely with an increase in *α*-helical secondary structure [38]. This relationship was speculated from the IR data. Antimicrobial peptides (AMPs) employ diverse mechanisms to disrupt cell membranes, making them effective against a wide range of pathogens. Their amphipathic structure allows them to interact with microbial membranes selectively. AMPs can insert themselves into the lipid bilayer, creating pores and increasing membrane permeability. This disrupts the electrochemical balance, leading to ion leakage and cell death [39]. Some AMPs target specific membrane regions, such as lipid rafts or lipopolysaccharides in Gram-negative bacteria, weakening structural integrity. Additionally, certain AMPs induce the generation of reactive oxygen species within microbial cells, causing damage to lipids, proteins, and DNA. Importantly, AMPs often exhibit selectivity for microbial membranes due to differences in composition compared to mammalian cell membranes, minimizing harm to host cells while effectively combating pathogens [40, 41].

The inhibitory effects of the peptide extract on various bacteria were evaluated by determining the minimum bactericidal concentration (MBC), as shown in Table 1. MBC represents the lowest concentration of the extract that kills 99.9% of the bacterial inoculants after 24-hour incubation at 37°C. Determining the MBC or MFC and calculating the MBC or MFC to MIC ratio provide an avenue for gaining a comprehensive understanding of the antimicrobial's effectiveness against a specific pathogen. Thus, to characterize the antimicrobial activity, the MBC to MIC ratio was calculated. A ratio of MBC/MIC ≤2 indicates a bactericidal effect, while a ratio ≥4 suggests a bacteriostatic effect. In our study, the peptide extract exhibited a bacteriostatic effect at the MIC for all microorganisms tested. However, above the MIC, a bactericidal effect was observed. The concentration of antimicrobial peptides plays a crucial role in their activity. At lower peptide : lipid ratios, cationic antimicrobial peptides remain associated with the membrane, aligned parallel to the lipid bilayer interface. As the peptide : lipid ratio increases, peptides can aggregate or reorient within the membrane, leading to membrane disruption and microbial death. Processes, such as ion channel formation, transmembrane pore formation, and membrane rupture, are more prominent at higher peptide concentrations [42].

The World Health Organization designates antibiotic-resistant *P. aeruginosa* as a top-priority pathogen due to its versatility and adaptability, enabling it to cause various infectious diseases. *Pseudomonas aeruginosa* stands out among other bacterial strains due to its distinct characteristics such as its ability to form biofilms, utilize quorum sensing, and employ efflux pumps among others, all of which enhances its pathogenic potential [43]. Biofilm-forming bacteria generally display broad-spectrum resistance to various antimicrobial agents and thus make it more challenging to eliminate biofilm-associated infections [32]. It is well documented that quorum sensing and biofilm formation are integral processes that modulate the social behavior of bacterial [44]. Biofilm formation in the Gram-negative microbe *P. aeruginosa* is under the control of the regulatory genes of quorum sensing (QS), and compounds that possess antiquorum sensing properties are routinely examined as prospective antibiofilm agents. These compounds are thought to interfere in the QS process and therefore inhibit the production of various virulence factors. Some commercially available anti-QS compounds have recently been shown to enhance the susceptibility of bacterial biofilm to various antimicrobial agents, both in *in vitro* and *in vivo* experiments [45]. Several AMPs have demonstrated significant potential as prospective treatments in the management of infectious diseases, with a number of them currently undergoing advanced stages of clinical trials [19, 46]. The strain of *P. aeruginosa* utilized in this investigation exhibited a remarkable ability to form biofilms. The production of pyoverdine and pyocyanin (both virulence factors) plays a crucial role in QS, a process that triggers biofilm formation. For QS research, the reference strain *P. aeruginosa* PA01 is renowned for its capacity to produce pyoverdine and pyocyanin when exposed to external AHL and is extensively employed.

**Figure 9:**
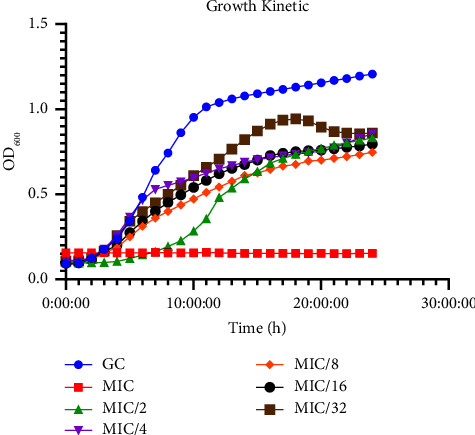
Growth curve of *Pseudomonas aeruginosa* in the absence (GC) and presence of varying concentrations of crude peptide (MIC, 1/32 MIC).

The peptide extract derived from *P. vulgaris* was assessed for its ability to inhibit the growth of *P. aeruginosa*, considering its antimicrobial properties. The MIC (1.25 mg/mL) of the peptide extract was determined to completely hinder bacterial growth, consequently preventing the formation of biofilms and the expression of virulent factors. Considering biofilm formation and production of virulence factors are dependent on bacteria quorum size, it was crucial to demonstrate that sub-MIC (0.625, 0.313, 0.156, 0.078, and 0.039) mg/mL concentrations of peptides did not hinder bacterial growth. Thus, the growth kinetics of bacteria were monitored by measuring the OD_600_. The absorption levels at OD_600_ showed no significant difference between the untreated group (control) and the cells treated with sub-MIC concentrations of peptides. While the rate of growth varied, the OD600 after 24 hours demonstrated that bacterial growth was unaffected (Figure 9) and quorum sizes could be reached. This suggests that any effect on any of the processes tested is due to the peptide extract interference in important cellular processes, rather than bacterial growth inhibition.

The impact of sub-MIC doses of the peptide mixture on the modulation of biofilm formation in *P. aeruginosa* was assessed, resulting in a biofilm inhibition rate ranging from 18% to 62%. Analysis of the data indicated that peptide concentrations within the range of the MIC, 1/2 MIC, and 1/4 MIC are necessary to achieve approximately 50% biofilm inhibition. It has been proposed that antibiofilm peptides operate by preventing microbial adhesion to surfaces, eliminating early surface colonizers, targeting preexisting biofilm-associated cells, and interfering with the microbe's quorum sensing mechanisms [3]. It is therefore likely that the peptide mixture derived from *P. vulgaris* might disrupt QS in *P. aeruginosa*, leading to its antibiofilm effects.

As quorum sensing (QS) in *P. aeruginosa* plays a role in regulating the expression of virulence factors like pyocyanin and pyoverdine, the peptide extract is expected to interfere with the production and expression of these harmful factors. Consequently, we examined the levels of pyocyanin and pyoverdine in the presence and absence of sub-MIC concentrations of the peptide extract. The presence of the peptide extract led to a reduction in pyoverdine levels, with inhibitory effects showing a clear dose-dependent pattern. At 1/2 MIC, pyoverdine production was inhibited by more than 44%. Similarly, pyocyanin production was inhibited by 81% at 1/2 MIC concentration. These findings demonstrate that the peptide mixture derived from *P. vulgaris* does indeed impede the production of virulence factors, using the same mechanism employed for biofilm inhibition. The probable mechanism of inhibition by the crude extract is interfering with the membrane of the bacteria by perturbing it. Such peptides have a strong affinity for the lipopolysaccharide groups that are the major components of bacterial cell membrane, and this leads to pore formation within the membrane, ultimately exposing intracellular organelles into the extracellular environment. On the other hand, biofilm inhibition is caused by the crude extract binding to the receptors responsible for biofilm formation, such as PqsR and RhlR [9, 26]. Pyoverdine, a fundamental siderophore produced by *P. aeruginosa*, serves the dual function of sequestering iron from host depots and acting as a QS signaling molecule. When pyoverdine is bound to iron, it interacts with the *P. aeruginosa* cell receptor FpvA, forming a complex that subsequently interacts with the antisigma factor FpvR. This interaction leads to the upregulation of exotoxin A, an endoprotease, as well as the upregulation of pyoverdine itself. Pyocyanin, on the other hand, induces oxidative stress in the host and stimulates the secretion of airway mucus [3, 19, 47]. All these virulent factors, including pyocyanin and pyoverdine, play crucial roles in the development and maintenance of biofilms, significantly contributing to the destructive nature of *P. aeruginosa* infections.

The peptide extract derived from *P. vulgaris* exhibits the ability to hinder the formation of biofilms in *P. aeruginosa* and also suppresses the expression of virulence factors. This indicates that the extract's mode of action involves targeting a shared factor involved in these processes. Since quorum sensing (QS) governs all these activities in *P. aeruginosa*, it is likely that the peptide extract interferes with cell-to-cell communication mediated by QS. There are very few drugs available that can effectively inhibit both quorum sensing and biofilm formation. The discovery of compounds and extracts that can perform these dual functions reduces the likelihood of antimicrobial resistance and offers a convenient approach for controlling pathogenic microorganisms. The promising results demonstrated by the peptide mixture from *P. vulgaris* in this regard present an opportunity for the development of innovative therapeutics that specifically target pathogenic bacteria.

## 5. Conclusion

In summary, the study findings indicate that the peptide extract obtained from *P. vulgaris* exhibits potent antimicrobial activity with a broad spectrum. At the minimum inhibitory concentrations (MICs), the extract demonstrates bacteriostatic effects. Furthermore, the study demonstrates the ability of the crude peptide extract to inhibit biofilm formation and suppress the expression of virulence factors, such as pyocyanin and pyoverdine, in *P. aeruginosa*. These results suggest that the crude peptide extract from *P. vulgaris* has the potential to serve as a valuable source for the development of new antimicrobial agents. With this, novel peptides and peptidomimetics can be isolated and design using isolated peptides from *P. vulgaris* crude. Other applications include the design of nanoparticles with effective antimicrobial activity.

## Figures and Tables

**Table 1:** MIC, MFC, MBC, microbistatic, and microbicidal effects of peptide extract.

Organism (gram status)	MIC (mg/mL) extract	MBC (mg/mL) extract	MBC/MIC	Effect	MIC (*μ*g/mL) gentamicin
*Candida albicans* (*fungi*)	1.25	>2.5^*∗*^	>2^*∗∗*^	STATIC	8.00^*∗∗∗*^
*Staphylococcus aureus* (*+*)	2.50	>2.5	>1	STATIC	12.50
*Pseudomonas aeruginosa* (*−*)	1.25	>2.5	>1	STATIC	6.25
*Escherichia coli* (*−*)	2.50	>2.5	>2	STATIC	12.50
*Enterococcus faecalis* (*+*)	2.50	>2.5	>2	STATIC	6.25

^*∗*^For *C. albicans*, MFC was determined. ^*∗∗*^The ratio determined is MFC/MIC. ^*∗∗∗*^Fluconazole was used for *C. albicans*.

## Data Availability

All data generated or analyzed during this study are included in this published article.
